# Temporal Variation of Phenolic and Mineral Composition in Olive Leaves Is Cultivar Dependent

**DOI:** 10.3390/plants9091099

**Published:** 2020-08-27

**Authors:** Igor Pasković, Igor Lukić, Paula Žurga, Valerija Majetić Germek, Mia Brkljača, Olivera Koprivnjak, Nikola Major, Kristina Grozić, Mario Franić, Dean Ban, Šime Marcelić, Smiljana Goreta Ban

**Affiliations:** 1 Department of Agriculture and Nutrition, Institute of Agriculture and Tourism, K. Huguesa 8, 52440 Poreč, Croatia; paskovic@iptpo.hr (I.P.); nikola@iptpo.hr (N.M.); grozic@iptpo.hr (K.G.); mario@iptpo.hr (M.F.); dean@iptpo.hr (D.B.); smilja@iptpo.hr (S.G.B.); 2 Centre of Excellence for Biodiversity and Molecular Plant Breeding, Svetošimunska 25, 10000 Zagreb, Croatia; 3 Teaching Institute of Public Health Primorsko-goranska County, Krešimirova 52a, 51000 Rijeka, Croatia; paula.zurga@zzjzpgz.hr; 4 Faculty of Medicine, Department of Food Technology and Control, University of Rijeka, Brace Branchetta 20, 51000 Rijeka, Croatia; valerija.majetic@medri.uniri.hr (V.M.G.); olivera.koprivnjak@medri.uniri.hr (O.K.); 5 Faculty of Food Technology and Biotechnology, University of Zagreb, Pierottijeva 6, 10000 Zagreb, Croatia; miabrkljaca@gmail.com; 6 Department of Ecology, Agronomy and Aquaculture, University of Zadar, Mihovila Pavlinovića bb, 23000 Zadar, Croatia; simemarcelic@unizd.hr

**Keywords:** abiotic stress, antioxidant activity, nutrients, *Olea europaea* L., oleuropein, sampling time

## Abstract

In order to investigate the potential of various olive cultivars and leaf sampling times for phytochemical farming practice in Croatia, phenolic and mineral composition was determined in olive leaves of four Croatian cultivars and Italian cultivar Leccino collected at three occasions, in October 2017, January 2018, and March 2018. Istarska bjelica turned out to have the largest phytochemical potential among the investigated cultivars due to steady high oleuropein concentrations found in its leaves. The concentration of main phenolic components in Istarska bjelica leaves changed only slightly during the sampling period, suggesting the possibility of its higher capability for low air temperatures stress resistance and different metabolic response compared to the other studied cultivars. Low air temperatures increased the oleuropein level and antioxidant activity in leaves of Leccino, Oblica, Levantinka, and Drobnica cultivars, which may be of crucial phytochemical farming interest. Each of the investigated olive cultivars was characterized by a specific leaf mineral nutrient composition, which could have had a specific role in their interplay with phenols.

## 1. Introduction

Olive tree (*Olea europaea* L.) is a traditional and economically the most important fruit tree of the Mediterranean [1]. It is well known for its capacity of producing organic matter with a high proportion of phenolic compounds [2]. In the search for bioactive compounds that can have significant role as nutraceuticals, as well as biological/medical agents, olive byproducts are known to be a rich source [3]. Among them, olive leaves (OL), traditionally used as a folk medicine remedy, have recently come into focus because of their proven beneficial effects on human health [4], which is mainly due to their abundance in phenols [5]. Thus, although olive is primarily grown for the production of olive oil and table olives, OL is recently being highly requested olive byproduct in the global market as a raw material for the extraction of valuable phytochemicals, mostly phenols.

Phenols, as one of the mayor groups of secondary metabolites in olive, are important because of their involvement in the plant response against biotic and abiotic stressors [3]. Beside phenols, which are common constituents of various plant species, olive phenols include the group of secoiridoids, which are exclusive for the Oleaceae family [4]. Secoiridoids such as oleuropein and its precursor hydroxytyrosol are considered the main phenolic compounds in OL [6] with proven anticarcinogenic, anti-inflammatory, and antimicrobial properties [7,8]. Besides them, other relatively abundant phenols in OL are flavone-7-glucosides of luteolin and apigenin, as well as verbascoside, which is a conjugated glucoside of hydroxytyrosol and caffeic acid [6].

Impact of the plant mineral status on its phenolic profile is generally well known [9]. Olive cultivar as well as sampling/harvest time can both have a significant effect on OL phenolic compounds [3] and mineral content [10,11]. However, other studies show that while the content of oleuropein, as the main OL secoiridoid, can be markedly modified due to the genetic background, variations attributed to the collecting period can be insignificant [2]. Despite some valuable published data, there is still a relatively large lack of information that would provide an integrative perspective on cultivar and sampling time interaction and its effects on both phenolic and mineral content in OL.

Different autochthonous olive cultivars with specific traits are grown in Croatian orchards, resulting in high quality olive oil products. In contrary to the research done up to date on olive oil [12,13], there is a lack of comprehensive studies about the quality and potential of OL of the main Croatian olive cultivars as a source material for the production of valuable phytochemicals such as phenols. In Mediterranean countries OL byproduct can be sustainably collected within the autumn harvest or spring pruning time. In such olive production practices, with defined cultivars and sampling times, additional economic income for local farmers can be planned and achieved.

For all the above reasons, this study aimed to investigate the temporal changes of the contents of minerals and phenols, as well as antioxidant capacity, in OL of four major Croatian autochthonous cultivars (Drobnica, Istarska bjelica, Lastovka, and Oblica) and Italian Leccino as the most common and widespread allochthonous olive cultivar in newly planted Croatian orchards [14]. The main hypothesis was that by identifying the most suitable cultivars and optimal sampling periods for obtaining OL richer in these valuable components more efficient OL phytochemical farming strategies might be planned. Although set on a particular location, it was assumed that the approach and the results obtained in this study could serve for developing a universal model of sustainable phytochemical farming applicable in other olive-growing regions worldwide.

## 2. Results

**Figure 1:**
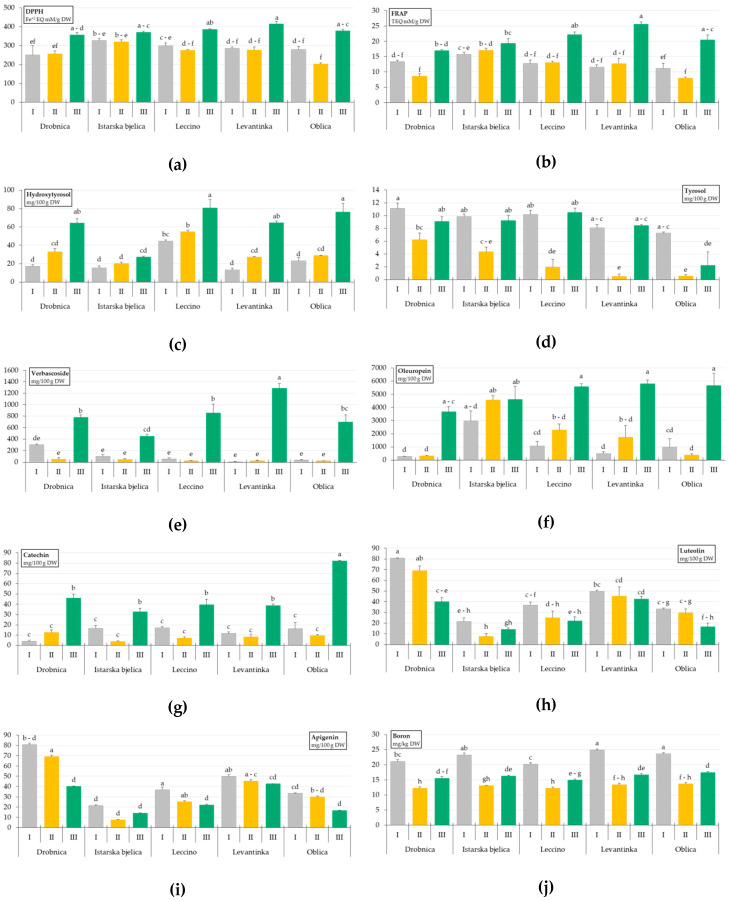
Multiple comparisons of the effects of cultivar × sampling time combinations on the antioxidant activity determined by 2,2-diphenyl-1-picrylhydrazyl (DPPH) and the ferric reducing ability of the plasma (FRAP) assays (**a**,**b**), and concentrations of particular phenols (**c**–**i**) and boron (**j**) in leaves of five olive cultivars (Drobnica, Istarska bjelica, Leccino, Levantinka, and Oblica) collected at different sampling times (I—October 2017, II—January 2018, and III—March 2018). Different superscript lowercase letters represent statistically significant differences between mean values at *p* < 0.05 obtained by a one-way ANOVA and Tukey’s test.

The values of antioxidant activity and dry weight (DW) concentrations of phenolic compounds in the leaves of different olive cultivars are shown in Table 1 and Table 2, while the DW concentrations of leaf macro and micronutrients are presented in Table 3. All significant cultivar × sampling time interactions (Cv. × ST) are shown in Figure 1a–j.

Regarding cultivar as a main factor, the antioxidant activity obtained by the 2,2-diphenyl-1-picrylhydrazyl (DPPH) assay was higher in Istarska bjelica compared to Drobnica and Oblica leaves, while when considering the ferric reducing ability of the plasma (FRAP) assay results Istarska bjelica, Leccino, and Levantinka had higher values than Drobnica and Oblica (Table 1). As far as sampling time was concerned as a main factor, after similar values obtained at ST I and ST II, an increase of antioxidant activity was observed at ST III. Cultivar and ST factors significantly interacted and multiple comparisons revealed a similar pattern of dynamic changes during time for each cultivar, with higher values at ST III compared to the other two sampling times. An exception was noted for cv. Istarska bjelica whose leaves had steadier values of antioxidant activity, evaluated by the both assays, through the experiment (Figure 1a,b).

Leccino leaves were characterized by the highest concentration of hydroxytyrosol (Table 1). Hydroxytyrosol levels gradually increased from ST I to ST III, and all the cultivars except Istarska bjelica reached the highest concentration in March 2018 at ST III (Figure 1c).

Tyrosol was found in higher concentration in Drobnica and Istarska bjelica leaves than in Levantinka and especially Oblica leaves, which contained the lowest concentration (Table 1). Although the Cv. × ST interaction was significant for this phenol, all the cultivars exhibited a similar behavior along the sampling period, with a decrease in concentration at ST II with respect to ST I, followed by an increase at ST III (Figure 1d).

The leaves of the Levantinka cultivar were characterized by the highest concentration of verbascoside, although not statistically different than those found in Drobnica and Leccino leaves (Table 1). The concentration of verbascoside at ST I and ST II was relatively similar, but increased drastically at ST III (Table 1, Figure 1e).

The highest oleuropein (Table 1) concentration was determined in Istarska bjelica leaves compared to all the other Croatian cultivars studied. Its levels were the highest in March (ST III) with the concentration average of 5061.22 mg/100 g DW. The cultivar × sampling time (Cv. × ST) interaction, however, revealed different oleuropein formation and accumulation dynamics between the observed cultivars. In Drobnica, Leccino, Levantinka, and Oblica leaves oleuropein dynamics followed the same pattern as mentioned previously for ST as a main factor, peaking in March. On the contrary, Istarska bjelica did not show any significant difference in the oleuropein level through all the STs (Figure 1f). The pattern of oleuropein accumulation coincided with the previously described changes in the antioxidant activity determined for OL of the investigated cultivars.

Oblica leaves contained the highest catechin concentration, while the highest rutin concentration was determined in Istarska bjelica leaves compared to all the other Croatian cultivars studied. The lowest concentration of rutin was found in Levantinka and Oblica leaves. Luteolin-7-O-glucoside was found in higher concentration in Levantinka than in Drobnica, Istarska bjelica, and Oblica leaves, while Leccino leaves were characterized by the highest concentration of apigenin-7-O-glucoside (Table 2). Rutin levels did not differ between the sampling times. Catechin and luteolin-7-O-glucoside followed a pattern already noted for the other phenols, with similar levels at ST I and ST II, and the highest concentration reached at ST III (Figure 1g). The concentration of apigenin-7-O-glucoside was higher at ST I and ST III than at ST II.

The highest concentration of luteolin was found in Drobnica, while apigenin was most abundant in Leccino and Levantinka leaves (Table 2). Istarska bjelica contained the lowest concentrations of these flavonoids. Unlike most of the other investigated phenols, luteolin and apigenin concentration mostly decreased through the sampling period, with the lowest concentrations found at ST III. Again, Istarska bjelica leaves showed different behavior with only slight changes in the concentrations along all the three STs. Changes in Oblica leaves were also not statistically different (Figure 1h,i).

Oblica leaves had higher phosphorous (P) concentration compared to Istarska bjelica, as well as the highest potassium (K) concentration compared to all the other cultivars (Table 3). Drobnica leaves were characterized by a higher calcium (Ca) concentration with respect to Leccino, Levantinka, and Oblica cv. leaves. Istarska Bjelica contained higher magnesium (Mg) concentration compared to Drobnica. The level of iron (Fe) was the lowest in Drobnica and Levantinka leaves, while the concentration of zinc (Zn) was higher in Oblica than in Leccino and Levantinka leaves. Higher concentration of manganese (Mn) was found in Leccino than in Istarska bjelica, Levantinka, and Oblica leaves. Copper (Cu) was found in higher concentration in Istarska bjelica than in Leccino leaves, while the concentration of boron (B) was higher in Istarska bjelica, Levantinka, and Oblica than in Drobnica and Leccino leaves.

The concentrations of P, Mg, and Zn followed a similar pattern of accumulation during the period of sampling, with somewhat lower values found at ST I (Table 3). Potassium, Fe, Cu, and B were found in the highest concentrations at ST I and then decreased at ST II and/or ST III. The concentration of Mn also decreased at ST II in relation to ST I, but increased again at ST III matching the level found at the beginning of the observation period. An increase at ST III was also noted for the concentration of B, although it did not reach initial ST I concentration as in the case of Mn (Table 3). In the case of B a significant Cv. × ST interaction was detected, with a higher concentration at ST I found in Levantinka and Oblica compared to Drobnica and Leccino leaves (Figure 1j).

Principal component analysis (PCA) analysis, including only variables with a significant Cv. × ST interaction, revealed three eigenvalues with values higher than 1 (Table 4). The first three principal components (PC1 55.42%, PC2 18.21%, and PC3 11.65%) accounted for 85.29% of the total variability (Table 4). PC1 correlated with FRAP, DPPH, oleuropein, and verbascoside content, while PC2 correlated with tyrosol and B. Negative PC1 values indicated that cultivars Leccino, Levantinka, and Oblica were connected with higher FRAP, DPPH, oleuropein, and verbascoside concentrations at ST III. Positive PC2 values confirmed the relationship between Drobnica and higher tyrosol and B leaf concentration at ST I, while an opposite trend was noted for the Leccino cultivar at ST II. On the other hand, PC3 values indicated the association of higher leaf concentration of luteolin with the Drobnica cultivar at ST II, as well as the lowest luteolin concentration with Istarska bjelica leaves collected at ST I and ST II.

**Figure 2:**
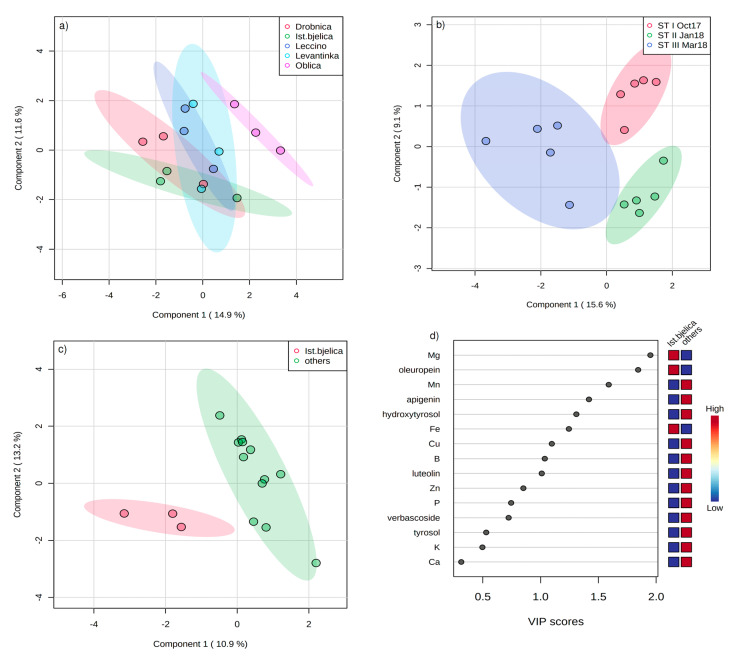
Separation of (**a**) cultivars (Drobnica, Istarska bjelica, Leccino, Levantinka, and Oblica) and (**b**) sampling times (ST I October 2017, ST II January 2018, and ST III March 2018) based on the content of phenols and mineral nutrients in olive leaf by partial least squares-discriminant analysis (PLS-DA); (**c**) separation of Istarska bjelica cultivar from the other four investigated cultivars by PLS-DA; and (**d**) phenols and minerals with the highest variable importance in projection (VIP) scores for the separation of Istarska bjelica cultivar from the other four investigated cultivars by PLS-DA.

Separation of the cultivars and sampling times based on the content of phenols and minerals in OL by partial least squares-discriminant analysis (PLS-DA) is shown in Figure 2a,b. Phenols and minerals with the highest variable importance in projection (VIP) scores for the differentiation according to the cultivar were, in decreasing order, B, tyrosol, luteolin, K, Cu, Ca, hydroxytyrosol, rutin, P, and luteolin-7-O-glucoside, while those with the highest VIP scores for the differentiation based on the sampling time were verbascoside, oleuropein, B, hydroxytyrosol, Zn, Mg, luteolin, DPPH, tyrosol, and luteolin-7-O-glucoside (data not shown). Istarska bjelica cultivar was successfully differentiated from all the other cultivars grouped in a single group, and the variables with the highest VIP scores were Mg, oleuropein, Mn, apigenin, hydroxytyrosol, Fe, Cu, B, luteolin, and Zn (Figure 2c,d).

## 3. Discussion

Plant phenols can be divided into two groups based on their origin, those that are naturally synthesized during normal plant development and those that have a role in the plant’s response to different stress factors [15]. Olive cultivars may have a different response to biotic or abiotic factors, consequently leading to distinct OL phenolic profiles and antioxidative activity [3]. Different leaf nutrient status was previously recorded for various olive cultivars grown in the same environment [11], which was probably linked to the differences in nutrient uptake and translocation between olive cultivars [16]. Furthermore, variation in OL nutrient status, depending on the sampling time, may have a major impact on OL phenolics [17,18], since nutrient availability is essential for providing co-factors for many enzymes of the phenolic compounds biosynthesis pathways [9].

For olive cultivars oleuropein can be considered as one of the main elements for determining the recovery capacity and resistance to freezing temperatures [19]. Among the cultivars chosen for this experiment, Leccino, Istarska bjelica, and Oblica have been previously described as tolerant to low temperatures [20,21,22,23], Levantinka was considered a sensitive one [22,23], while the information about Drobnica low temperature tolerance were contradictory [24,25].

**Figure 3:**
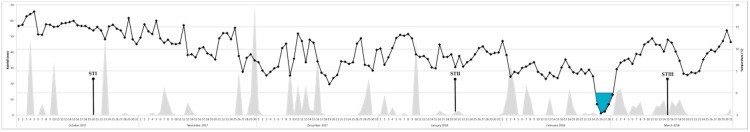
Average daily temperatures (°C) and rainfall (mm) measured in the sampling period from the beginning of October 2017 until the end of March 2018 on the experiment location with indicated sampling times (STI–STIII). Negative temperatures are marked by the blue area.

In this study, antioxidant activity (AA), oleuropein, and hydroxytyrosol concentration changed only slightly in leaves of Istarska bjelica through the period from October 2017 (ST I) to March 2018 (ST III). However, all the other studied cultivars have shown the highest DPPH (Figure 1a), oleuropein (Figure 1f), and hydroxytyrosol (Figure 1c) levels at ST III. Fifteen days prior to ST III the average daily air temperatures have reached a minimum ranging from −4.6 to −2.5 °C during three days, with an absolute minimum of −6.4 °C (Figure 3), suggesting that stress caused by low air temperature led to a significant increase of oleuropein concentration in Leccino, Oblica, Drobnica, and Levantinka leaves observed at ST III (Figure 1f). Ortega-García and Peragón [19] found that in moderately and heavily cold stressed OL collected 15 days after air temperature fell below −7 °C, the hydroxytyrosol level was lower and the oleuropein level was higher compared to leaves of the same cultivar that have not been stressed. The same authors concluded that since high oleuropein concentration is probably closely related to higher antioxidant capacity, by lowering the level of cellular reactive oxygen species (ROS) during cold stress, this compound may offer protection against oxidative damage induced by freezing. Gubanova and Paliy [26] reported that the total content of phenolic compounds in leaves of frost-resistant olive cultivars remained unchanged under the influence of temperatures close to absolute minimum value of −15 °C. A similar response observed in the case of the majority of the most abundant phenols and antioxidant activity in Istarska bjelica leaves confirmed the practical knowledge about its high tolerance to low temperatures [21,23]. It is possible that already high oleuropein concentration in Istarska bjelica leaves at ST I and ST II was sufficient to provide adequate protection against possible oxidative damage induced by low temperatures at ST III (Figure 1f), and the defense mechanisms activated were not the same as in the case of the other cultivars. Constantly high oleuropein level in leaves of Istarska bjelica during the whole sampling period clearly pointed to the possibility that this is a genetically predetermined feature of this cultivar. Distinctiveness of Istarska bjelica was also confirmed by PLS-DA, which successfully differentiated it from the other cultivars, with oleuropein as a phenol with the highest VIP score (Figure 2c,d).

Strong negative correlations were determined between the concentration of K and DPPH (*p* < 0.001, *r* = −0.65), FRAP (*p* < 0.001, *r* = −0.62), and oleuropein (*p* < 0.001, *r* = −0.67) in OL, respectively, for the whole dataset (Table S1). When particular cultivars were observed, all of them except Istarska bjelica showed a strong negative correlation between K and oleuropein concentration (Table S2). Wang et al. [27] pointed out that significant negative correlation could be observed between the level of frost damage and K concentration in OL due to the fact that high K concentrations may participate in lowering the freezing point of plant cell solution. However, in our experiment despite relatively low K concentration in leaves of all the studied cultivars, no visible damage caused by low air temperatures was observed at ST III. In previous studies contradictory data on this matter were found. Nguyen et al. [28] found that total phenolic concentration was increased at a higher K rate in basil, while Hafsi et al. [29] noted its increase under K deficiency in *Sulla carnosa* plants. Additionally, Sun et al. [30] reported that K suppresses the accumulation of phenols in blueberries. In this study, leaves from all the observed cultivars, except Oblica, were characterized by relatively low K concentration (Table 3) according to Therios [31]. A significant decrease of K concentration from ST I to ST II was observed in this study (Table 3). Similar fluctuations in K concentration in leaves of cv. Picual during an “off” season have been reported by Fernandez-Escobar et al. [10].

Mineral status of plants is essential in providing co-factors for many enzymes of the phenylpropanoid and flavonoid pathway. Magnesium (Mg) and Mn cations are crucial for ensuring the functioning of phenylalanine ammonia-lyase (PAL), one of the key enzymes responsible for phenol biosynthesis [9]. Olive leaves of all the investigated cultivars were characterized by Mg and Mn concentrations (Table 3) above the mineral deficiency levels of 8 g/kg and 20 mg/kg established by Connor and Fereres [20], respectively, suggesting their optimal nutrient status. A difference in Mg leaf concentration was noted only between Istarska bjelica and Drobnica cultivars (Table 3). The concentration of Mg in leaves correlated positively with oleuropein (*p* < 0.01, *r* = 0.44) and negatively with luteolin concentration (*p* < 001, *r* = −0.63; Table S1). Thus, Istarska bjelica, whose leaves contained the lowest luteolin concentration, was the most abundant in Mg. This result was confirmed by PCA, which pointed to lower luteolin content of Istarska bjelica compared to all the other cultivars at ST I and ST II (Table 4), as well as by PLS-DA where Mg was a variable with the highest VIP score for the differentiation of Istarska bjelica from the other cultivars (Figure 2c,d). Increment of macronutrient foliar concentrations was previously reported to reduce luteolin concentration in artichoke and cardoon leaves [32].

Combined foliar application of Mg, Mn, and B resulted with a significant decrease of flavonoid content in OL [33]. In contrast, in this study strong positive correlation was revealed between Mn and tyrosol (*p* < 0.001, *r* = 0.58), as well as apigenin-7-O-glucoside concentration (*p* < 0.001, *r* = 0.62), respectively (Table S1). Leccino cultivar leaves contained the highest concentration of apigenin-7-O-glucoside and Mn in relation to all the other investigated cultivars (Table 2 and Table 3).

Verbascoside, as the second most abundant OL phenolic component determined in this study, was found in the highest concentration at ST III in OL of all the cultivars (Figure 1e). Negative correlation between Fe and verbascoside concentration was recorded (*p* < 0.001, *r* = −0.56; Table S1). Difference in the concentration of Fe in OL of various cultivars was previously reported by a number of authors [11,34]. Cultivar × ST interaction showed that Levantinka had statistically the highest verbascoside concentration at ST III (Figure 1e), although all the other investigated cultivars followed the same pattern, even Istarska bjelica who differed based on the temporal variation of some other phenols, such as oleuropein (Figure 1f). As the phenolic profile differs among olive cultivars, with oleuropein and verbascoside as the main components, small-fruit olive varieties were previously shown to contain tissues with high levels of oleuropein and low levels of verbascoside, while the opposite was noted for large-fruited cultivars [31]. In this study, a strong positive correlation was found between oleuropein and verbascoside concentrations in the whole dataset (*p* < 0.001, *r* = 0.73), particularly for Drobnica as a small fruit cultivar (*p* < 0.001, *r* = 0.93) and Oblica as a large fruit cultivar (*p* < 0.001, *r* = 0.97). Interestingly, among middle size fruit cultivars, oleuropein and verbascoside concentrations did not correlate in Istarska bjelica OL, while Leccino (*p* < 0.01, *r* = 0.86) and Levantinka (*p* < 0.001, *r* = 0.86) showed a very strong correlation (Table S2).

Tyrosol, as a major phenolic precursor involved in the oleuropein biosynthesis [35,36], was apparently connected to higher B concentration according to PCA (Table 4). In our previous study, B foliar fertilization has shown different effects through sampling time periods on OL tyrosol concentration [18]. Boron concentration was above the deficiency limit of 14 mg/kg DW as defined by Connor and Ferreres [20] in leaves of all the cultivars investigated in this study at ST I and ST III (Figure 1j). On the contrary, the B level in leaves of all the cultivars was below the deficiency threshold at ST II (Figure 1j). Karioti et al. [37] reported that B nutrient deficiency stress in OL results in higher levels of specific secoiridoids. Liakopoulos and Karabourniotis [38], however, in a field trial experiment revealed higher oleuropein concentration in B-sufficient in contrast to B-deficient OL. In this study no significant correlation was established between B and the oleuropein OL concentration.

The levels of all the other investigated minerals in OL were above the corresponding deficiency levels according to the literature [20,31], independent of the main factors or their interactions (Table 3). The P concentration found could have been considered as near or above the excess levels of >1.4 g/kg [31] or within the adequate level range of 1–3 g/kg [20], depending on the literature source.

## 4. Conclusions

The results obtained in this study showed significant differences in the concentration of many leaf constituents between the investigated olive cultivars. This is especially important in the case of the most abundant phenol oleuropein, which is among the principal carriers of OL antioxidant activity and largely determines the value of this byproduct as a source of valuable phytochemicals. From the practical and commercial point of view, Istarska bjelica turned out to have the largest potential for phytochemical farming among the investigated cultivars due to steady high oleuropein concentrations found in its leaves. The investigated cultivars were characterized by different temporal variations of phenolic content and antioxidant activity, but generally reached the highest levels at the spring sampling time, suggesting that the efforts made during pruning could be additionally valorized by exploitation of such leaves naturally enriched in valuable bioactive ingredients. The Istarska bjelica cultivar maintained relatively similar levels of the main phenolic OL components during the whole sampling period, suggesting the possibility of its high capability for low temperature stress resistance. As well, Istarska bjelica exhibited a unique pattern of correlation of phenols with OL mineral components, which indicated that its metabolic response to particular environmental factors might be different with respect to the other cultivars studied. Oleuropein concentration and antioxidant activity in Drobnica, Leccino, Levantinka, and Oblica leaves increased following exposure to low air temperatures, which may be of crucial interest for designing more efficient and sustainable phytochemical farming strategies. Each of the investigated cultivars seemed to be connected to a specific OL mineral nutrient composition, which could have had a specific role in their interplay with phenols. However, to draw more precise conclusions, specific influence of each or the combination of particular mineral nutrients on OL phenolic composition should be further confirmed through plant nutrition experiments. More successful PLS-DA differentiation of OL samples based on sampling time than on cultivar (with Istarska bjelica as an exception) confirmed that regardless of the differences among cultivars, particular biosynthetic pathways during olive plant development are common or at least analogous within the species. This also confirmed the important role of particular phenols and minerals in maintaining normal plant development and resistance to different agroclimatic conditions during the season.

## 5. Materials and Methods

### 5.1. Olive Leaves Sampling

The leaf samples were collected at the olive orchard near Linardići (45°04′25″/14°27′54″/60 m) at the island of Krk, Croatia. The orchard, with 150 olive trees (5 m × 5 m row arrangement), is planted in the North–South direction on *Terra rossa* soil [39] (Table S3) and located in the climate area, which is classified as Cfa according to Köppen [40].

At the sampling time the orchard was 8 years old and standard fertilization practice were applied each year [41]. At the pre-experiment period, in the autumn of 2016, 1 kg (NPK 7:20:30)/tree rate was applied and additionally 1 kg (KAN)/tree was incorporated in soil during the 2017 spring period. During the 2017 summer period each olive tree was irrigated with 50 L of rainwater every week. The same fertilization practice was repeated during the autumn of 2017.

Only well developed, healthy, equally conditioned trees of the four main Croatian olive cultivars (Istarska bjelica, Oblica, Lastovka, and Drobnica) and Italian cultivar Leccino were selected for the experiment. Each leaf sample comprised of 200 leaves from the middle portion of one year olive shoots, taken equally around the olive tree. Samples were collected at three sampling times, at the harvest period on 20 October 2017, during the winter dormancy period on 20 January, and at the pruning time on 15 March 2018. Set as random block design with three repetitions, with each of the 5 cultivars represented by 3 trees, the total number of trees in the experiment was 15.

The average daily temperatures and quantity of rainfall (Figure 3) were recorded on the nearest location (Krk, Croatia) and the data were obtained from the Croatian Meteorological and Hydrological Service.

All the samples, after each sampling time, were taken to the laboratory and carefully rinsed sequentially with tap water, 1% acetic acid solution with deionized water, and deionized water. Plant material was then air dried until constant mass and milled to fine powder [18].

### 5.2. Chemicals

Methanol (MeOH) and acetonitrile (AcN) were purchased from Merck (Darmstadt, Germany) and phosphoric acid from Sigma-Aldrich (St. Louis, MO, USA). Standards of apigenin, apigenin-7-O-glucoside, catechin, hydroxytyrosol, luteolin, luteolin-7-O-glucoside, oleuropein, rutin, tyrosol, and verbascoside were purchased from Extrasynthese (Genay, France). Deionized water was obtained by Siemens UltraClear (Siemens AG, München, Germany).

Multi-element standard solution from Perkin Elmer (NexION Setup Solution, Waltham, MA, USA) was used. Argon used to form plasma for the inductively coupled plasma mass spectrometer (ICP-MS) analysis was of purity 6.0 (Messer, Austria). Acetylene was supplied by Messer (Messer Croatia Plin d.o.o., Zaprešić, Croatia).

Deionized water was obtained by Siemens UltraClear (Siemens AG, München, Germany).

### 5.3. Measurement of the Total Antioxidant Capacity

Total antioxidant capacity was determined using an UV-VIS spectrophotometer (Model UV-1800, Shimadzu Corporation, Kyoto, Japan) by both the ability of each sample to scavenge the 2,2-diphenyl-1-picrylhydrazyl (DPPH) radical [42] and by the ferric reducing ability of the plasma assay (FRAP) [43]. DPPH radical scavenging activity was determined by analyzing a mixture of 1 mL of the sample with 2 mL of 0.1 mM DPPH radical at 517 nm after 30 min in darkness. The results were expressed as mM of Trolox equivalents per g sample DW. FRAP values were obtained by analyzing a mixture of 1 mL of sample with 2 mL of freshly prepared FRAP reagent at 593 nm after 4 min of reaction time. Results were expressed as mM of Fe^2+^ equivalents per g sample DW.

### 5.4. High Performance Liquid Chromatography

Phenolic compounds were determined by high-performance liquid chromatography (HPLC) using a Thermo Ultimate 3000 System, comprised of a degasser, a binary pump, an autosampler, a column oven, and an UV/Vis detector capable of simultaneous measurement at 4 different wavelengths (ThermoFisher Scientific, Waltham, MA, USA). Phenols from OL were extracted by a previously described procedure with minor modifications [44]. Air dried and finely ground olive leaves (500 mg) were extracted with 20 mL of methanol 80% (v/v) in an ultrasonic bath (frequency 35 kHz, power 125 140/560 W, Sonorex Digitec, Bandelin electronic, Berlin, Germany) for 20 min. An aliquot (14 mL) of the extract was centrifuged for 7 min at 4000 rpm and the supernatant was filtered through a 0.45 μm-pore cellulose acetate syringe filter. The separation of phenols was performed using a Lichrospher 100 RP-18 (250 mm × 4 mm, 5 µm) analytical column with a pre-column Lichrospher 100 (4 mm × 4 mm, 5 µm), both supplied by Agilent Technologies (Santa Clara, CA, USA). The analyses were performed at a constant temperature of 25 °C. The mobile phase consisted of (A) 0.2% phosphoric acid and (B) MeOH: AcN (1: 1). The chromatographic conditions were as follows: 10% B 0–0.5 min; 10%–16.5% B 0.5–25 min; 16.5%–30% B 25–80 min; 30%–100% B 80–95 min; 100% B 95–100 min; 100%–10% B 100–102 min; and 10% B 102–105 min, followed by equilibration time for 10 min. The flow rate was 0.8 mL/min. UV/Vis detection was set at 250 nm for luteolin-7-O-glucoside and oleuropein, 280 nm for apigenin-7-O-glucoside, catechin, hydroxytyrosol, and tyrosol, 305 nm for apigenin, caffeic acid, and verbascoside, and 370 nm for luteolin and rutin. Identification was performed by comparing retention times of the target compounds in the sample extracts with the retention times of pure standards. Quantification was done using the external standard method. The calibration curves for individual polyphenols were obtained by using five calibration levels made by appropriate dilutions of the stock standard solutions and calibration curves with *R*^2^ ≥ 0.999 were accepted for concentration calculation.

### 5.5. Elemental Analysis

Air dried and finely ground olive leaves (500 mg) were weighed in porcelain dishes and dry ashed at 550 °C for 8 h. The ash was dissolved in 5 mL of 0.6 M hydrochloric acid (Normapur, VRW International, Randor, PA, USA) with heating for 15 min at 60 °C. The solution was filtered over Munsell No. 388 filter paper in PE graduated tubes and diluted to 50 mL with deionized water [45].

Analyses of B, Cu, Mn, and Zn were performed using an inductively coupled plasma mass spectrometer (ICP-MS) NexION 300× (PerkinElmer Instruments, Waltham, MA, USA) equipped with an S10 autosampler. Multi-element solution (NexION Setup Solution, PerkinElmer, Waltham, MA, USA) was used as a tuning solution, covering a wide range of masses of the elements. The oxide ratio and double charged species were maintained below 0.03%. Calcium (Ca), Mg, K, and Fe were analyzed by a flame atomic absorption spectrometer (FAAS) PerkinElmer AAS800 (PerkinElmer Instruments, Waltham, MA, USA) using an acetylene-air oxidant. Phosphorous (P) was determined by using an UV-VIS spectrophotometer (Carry UV/Vis 50, Varian Inc., Palo Alto, CA, USA), following the method proposed by Miller [46]. Each calibration curve was obtained by using at least six (ICP-MS) or five calibration levels (FAAS, UV/Vis) made by appropriate dilutions of the multi-element standard solution with the same acid matrix. The calibration curves with *R*^2^ ≥ 0.999 were accepted for concentration calculation, with ranges suitable for the investigated analytes. A mean of five runs was obtained for each sample/element. Reagent blanks were prepared and determined in the same way as the samples.

### 5.6. Soil and Cultivar Characterization

The chemical properties of the selected soil are reported in Table S3 and were determined as described in Pasković et al. [47]. Characterization of Drobnica [14,24,25,48], Istarska bjelica [23], Leccino [14,23], Levantinka [14,23] and Oblica [14,21,23] cultivars is listed in Table S4.

### 5.7. Statistical Analysis

The experiment was set up as a random block design in three replications. A two-way analysis of variance (ANOVA) was performed for all the data, with cultivar and sampling time as the main factors. Multiple comparisons of means were based on a Tukey’s test at *p* ≤ 0.05. For principal component analysis (PCA) only principal components (PCs) with an eigenvalue >1.0 were selected, and in each of the selected PCs only variables with high factor loadings (within 10% of highest absolute value) were retained for observation [49]. ANOVA, post-hoc comparisons, and PCA were performed using Statistica v. 13.4 software (Tibco Software Inc., Palo Alto, CA, USA). Partial least squares-discriminant analysis (PLS-DA) was applied to find the most useful variables among the investigated phenolic compounds and minerals for the differentiation of OL samples based on cultivar and sampling time. PLS-DA was applied on auto-scaled data using MetaboAnalyst v. 4.0 created at the University of Alberta, Canada [50].

## Figures and Tables

**Table 1:** Antioxidant activity measured by the 2,2-diphenyl-1-picrylhydrazyl (DPPH) and the ferric reducing ability of the plasma (FRAP) assays and concentrations of simple phenols, phenolic acids, and oleuropein in leaves of five olive cultivars collected at different sampling times.

Source of Variation	Antioxidant Activity		Simple Phenols		Phenolic Acids	Secoiridoids
	DPPH	FRAP	Hydroxytyrosol	Tyrosol	Verbascoside	Oleuropein
	(mM/g DW TEQ)	(mM/g DW Fe^2+^EQ)	(mg/100 g DW)		(mg/100 g DW)	(mg/100 g DW)
**Cultivar (Cv.)**						
Drobnica	288.06 ± 23.00 ^b,c^	12.93 ± 1.24 ^b^	38.05 ± 7.15 ^b^	8.81 ± 0.84 ^a^	376.53 ± 108.24 ^a,b^	1411.77 ± 576.63 ^c^
Istarska bjelica	339.77 ± 8.99 ^a^	17.32 ± 0.75 ^a^	20.92 ± 1.89 ^c^	7.80 ± 0.93 ^a^	198.22 ± 64.96 ^c^	4041.88 ± 460.74 ^a^
Leccino	320.47 ± 17.48 ^a,b,c^	15.97 ± 1.60 ^a^	59.99 ± 6.01 ^a^	7.51 ± 1.47 ^a,b^	311.73 ± 143.32 ^a,b,c^	2976.18 ± 698.44 ^a,b^
Levantinka	325.66 ± 23.44 ^a,b^	16.59 ± 2.31 ^a^	34.91 ± 7.67 ^b^	5.67 ± 1.31 ^b^	440.23 ± 212.24 ^a^	2672.34 ± 846.91 ^b,c^
Oblica	286.93 ± 25.85 ^c^	13.17 ± 1.99 ^b^	42.76 ± 8.88 ^b^	3.33 ± 1.19 ^c^	252.09 ± 117.87 ^b,c^	2334.52 ± 899.47 ^b,c^
**Sampling time (ST)**						
I	288.84 ± 11.51 ^b^	12.90 ± 0.58 ^b^	22.71 ± 3.17 ^c^	9.29 ± 0.43 ^a^	99.68 ± 29.12 ^b^	1152.94 ± 313.54 ^b^
II	266.24 ± 11.03 ^b^	11.85 ± 0.95 ^b^	32.71 ± 3.21 ^b^	2.71 ± 0.68 ^c^	34.01 ± 6.73 ^b^	1847.85 ± 453.58 ^b^
III	381.45 ± 6.38 ^a^	20.84 ± 0.89 ^a^	62.56 ± 5.55 ^a^	7.88 ± 0.89 ^b^	813.60 ± 81.85 ^a^	5061.22 ± 330.98 ^a^
**Cv.**	***	***	***	***	***	***
**ST**	***	***	***	***	***	***
**Cv. × ST**	*	***	***	***	***	**

Results are expressed as means ± standard errors. Different superscript lowercase letters in a column represent statistically significant differences between mean values for each main effect at *p* < 0.05 obtained by obtained by a two-way ANOVA and Tukey’s test. Significance: ***—*p* < 0.001, **—*p* < 0.01, *—*p* < 0.05. DW—dry weight; TEQ—Trolox equivalents; EQ—equivalents.

**Table 2:** Concentrations of flavonoids in leaves of five olive cultivars collected at different sampling times.

Source of Variation	Flavonoids					
	Catechin	Rutin	Luteolin-7-O-Glucoside	Apigenin-7-O-Glucoside	Luteolin	Apigenin
	(mg/100 g DW)					
**Cultivar (Cv.)**						
Drobnica	21.01 ± 6.56 ^b^	66.61 ± 5.07 ^b^	346.66 ± 30.10 ^b,c^	34.64 ± 4.89 ^c^	63.29 ± 6.27 ^a^	6.76 ± 1.80 ^a,b^
Istarska bjelica	17.67 ± 4.36 ^b^	97.78 ± 6.56 ^a^	294.58 ± 15.20 ^c^	39.83 ± 3.13 ^c^	14.31 ± 2.44 ^d^	2.33 ± 0.28 ^c^
Leccino	21.24 ± 5.07 ^b^	70.68 ± 6.38 ^b^	401.33 ± 27.68 ^a,b^	85.47 ± 8.36 ^a^	27.95 ± 3.22 ^c^	9.38 ± 1.84 ^a^
Levantinka	19.57 ± 4.89 ^b^	44.43 ± 4.01 ^c^	445.36 ± 30.56 ^a^	60.22 ± 6.67 ^b^	45.89 ± 2.80 ^b^	8.48 ± 1.33 ^a^
Oblica	35.95 ± 11.74 ^a^	34.96 ± 2.86 ^c^	356.48 ± 30.63 ^b,c^	28.49 ± 2.33 ^c^	26.58 ± 2.85 ^c^	4.03 ± 0.83 ^b,c^
**Sampling time (ST)**						
I	13.19 ± 1.79 ^b^	65.17 ± 8.11	319.89 ± 16.51 ^b^	52.97 ± 6.65 ^a^	44.37 ± 5.48 ^a^	7.47 ± 1.24 ^a^
II	8.24 ± 1.04 ^c^	57.54 ± 5.98	354.28 ± 25.10 ^b^	37.60 ± 5.49 ^b^	35.32 ± 5.93 ^b^	8.83 ± 1.15 ^a^
III	47.83 ± 4.89 ^a^	65.97 ± 6.68	432.48 ± 21.83 ^a^	58.62 ± 7.35 ^a^	27.12 ± 3.40 ^c^	2.28 ± 0.30 ^b^
**Cv.**	***	***	***	***	***	***
**ST**	***	**n.s.**	***	***	***	***
**Cv. × ST**	***	**n.s.**	**n.s.**	**n.s.**	**	***

Results are expressed as means ± standard errors. Different superscript lowercase letters in a row represent statistically significant differences between mean values for each main effect at *p* < 0.05 obtained by a two-way ANOVA and Tukey’s test. Significance: n.s.—not significant, ***—*p* < 0.001, **—*p* < 0.01, *—*p* < 0.05. DW—dry weight.

**Table 3:** Concentrations of mineral nutrients in leaves of five olive cultivars collected at different sampling times.

	Macronutrients (g/kg DW)				Micronutrients (mg/kg DW)				
Source of Variation	P	K	Ca	Mg	Fe	Zn	Mn	Cu	B
**Cultivar (Cv.)**									
Drobnica	1.51 ± 0.09 ^a,b^	6.74 ± 0.49 ^b^	25.07 ± 1.55 ^a^	8.00 ± 0.43 ^b^	68.24 ± 3.54 ^c^	23.37 ± 0.68 ^a,b^	57.69 ± 4.46 ^a,b^	12.94 ± 2.31 ^a,b^	16.28 ± 1.33 ^b^
Istarska bjelica	1.36 ± 0.05 ^b^	5.28 ± 0.30 ^c^	19.59 ± 1.70 ^b^	10.14 ± 0.30 ^a^	83.15 ± 4.15 ^a,b^	23.09 ± 1.13 ^a,b^	50.31 ± 3.54 ^b^	16.19 ± 2.18 ^a^	17.52 ± 1.51 ^a^
Leccino	1.55 ± 0.05 ^a,b^	5.91 ± 0.32 ^b,c^	22.19 ± 1.05 ^a,b^	9.06 ± 0.19 ^a,b^	88.95 ± 5.28 ^a,b^	22.17 ± 0.64 ^b,c^	67.68 ± 6.25 ^a^	11.09 ± 1.73 ^b^	15.78 ± 1.18 ^b^
Levantinka	1.45 ± 0.04 ^a,b^	6.04 ± 0.36 ^b,c^	17.50 ± 1.15 ^b,c^	9.48 ± 0.54 ^a,b^	70.24 ± 5.20 ^c^	22.27 ± 1.21 ^b,c^	51.71 ± 2.63 ^b^	14.66 ± 1.55 ^a,b^	18.28 ± 1.72 ^a^
Oblica	1.67 ± 0.03 ^a^	7.85 ± 0.42 ^a^	11.45 ± 0.62 ^c^	9.45 ± 0.22 ^a,b^	74.63 ± 2.40 ^b,c^	25.85 ± 0.66 ^a^	46.74 ± 3.00 ^b^	13.39 ± 1.35 ^a,b^	18.20 ± 1.47 ^a^
**Sampling time (ST)**									
I	1.43 ± 0.06 ^b^	7.46 ± 0.33 ^a^	20.23 ± 1.96	8.53 ± 0.33 ^b^	85.87 ± 4.17 ^a^	22.09 ± 0.72 ^b^	63.75 ± 3.80 ^a^	19.94 ± 1.16 ^a^	22.58 ± 0.49 ^a^
II	1.61 ± 0.04 ^a^	6.36 ± 0.29 ^b^	19.29 ± 1.48	9.59 ± 0.31 ^a^	78.82 ± 3.11 ^a^	23.34 ± 0.85 ^a,b^	43.39 ± 2.06 ^b^	10.38 ± 0.71 ^b^	12.91 ± 0.22 ^c^
III	1.48 ± 0.04 ^a,b^	5.27 ± 0.23 ^c^	17.97 ± 1.07	9.56 ± 0.32 ^a,b^	66.43 ± 1.83 ^b^	24.62 ± 0.55 ^a^	57.34 ± 2.70 ^a^	10.64 ± 0.68 ^b^	16.14 ± 0.29 ^b^
**Cv.**	**	***	***	**	***	*	***	*	***
**ST**	*	***	**n.s.**	*	***	*	***	***	***
**Cv. × ST**	**n.s.**	**n.s.**	**n.s.**	**n.s.**	**n.s.**	**n.s.**	**n.s.**	**n.s.**	*

Results are expressed as means ± standard errors. Different superscript lowercase letters in a row represent statistically significant differences between mean values for each main effect at *p* < 0.05 obtained by a two-way ANOVA and Tukey’s test. Significance: n.s.—not significant, ***—*p* < 0.001, **—*p* < 0.01, *—*p* < 0.05. DW—dry weight.

**Table 4:** Parameters of principal component analysis (PCA) for the separation of olive leaves of five cultivars (Drobnica, Istarska bjelica, Leccino, Levantinka, and Oblica) collected at different sampling times (ST I—October 2017, ST II—January 2018, and ST III—March 2018) based on the values of antioxidant activity measured by the 2,2-diphenyl-1-picrylhydrazyl (DPPH) and the ferric reducing ability of the plasma (FRAP) assays, and the concentration of phenols and mineral nutrients as variables.

Statistic	PC1	PC2	PC3
*Eigenvalue*	*5.54*	*1.82*	*1.17*
*% variance*	*55.42*	*18.21*	*11.65*
*Cumulative %*	*55.42*	*73.63*	*85.29*
**Factor loading/eigenvector**			
*Variables*			
DPPH	**−0.945**	0.170	0.024
FRAP	**−0.945**	0.118	0.004
Hydroxytyrosol	−0.747	−0.284	−0.471
Tyrosol	−0.222	**0.881**	−0.088
Catechin	−0.825	−0.037	−0.143
Verbascoside	**−0.883**	0.153	−0.355
Oleuropein	**−0.956**	−0.151	0.155
Luteolin	0.492	0.403	**−0.695**
Apigenin	0.716	−0.135	−0.454
B	0.137	**0.832**	0.277
*Cases*			
Drobnica × ST I	2.055	**2.198**	−0.932
Drobnica × ST II	2.612	−0.543	**−1.767**
Drobnica × ST III	−2.143	0.305	−0.750
Istarska bjelica × ST I	0.064	1.469	**1.742**
Istarska bjelica × ST II	−0.286	−1.235	**1.908**
Istarska bjelica × ST III	−1.789	0.319	1.193
Leccino × ST I	1.282	0.803	−0.542
Leccino × ST II	1.173	**−2.113**	−0.418
Leccino × ST III	**−3.448**	0.094	−0.567
Levantinka × ST I	2.281	1.592	0.134
Levantinka × I ST II	1.769	−1.574	−0.286
Levantinka × ST III	**−3.743**	0.645	−1.193
Oblica × ST I	1.360	1.061	1.095
Oblica × ST II	2.477	−1.871	0.385
Oblica × ST III	**−3.665**	−1.150	−0.004

## Supplementary Materials

The following are available online at https://www.mdpi.com/2223-7747/9/9/1099/s1, Table S1. Correlation between antioxidant activity and the concentrations of selected phenolic compounds, macronutrients, and micronutrients in leaves of five olive cultivars collected at different sampling times; Table S2. Correlation between the concentrations of verbascoside, oleuropein, and potassium for Drobnica, Istarska bjelica, Leccino, Levantinka, and Oblica collected at different sampling times; Table S3: Chemical properties of the *Terra rossa* soil; Table S4. Description of olive cultivars included in the study.
