# Supplementary material for: Temporal Variation of Phenolic and Mineral Composition in Olive Leaves Is Cultivar Dependent
Source: Plants (Basel). 2020 Aug 27;9(9):1099. doi: 10.3390/plants9091099 (PMC7570285; doi:10.3390/plants9091099)
Supplement: Supplementary file 1 [file plants-09-01099-s001.pdf]

plants

MDPI

SUPPLEMENTARY MATERIAL

Table S1. Correlation* between antioxidant activity and the concentrations of selected phenolic compounds, macronutrients, and micronutrients in leaves of five olive cultivars collected at different sampling times.

Variables		Antioxidant activity		Simple phenols	Phenolic acids	Secoiridoids	Flavonoids		Micronutrients		Macronutrients	
		**DPPH**	**FRAP**	**Tyrosol**	**Verb**	**Ole**	**Api-7-O**	**Luteolin**	**Fe**	**Mn**	**K**	**Mg**
**DPPH**	*r*	1.000	0.9013	0.3449	0.7718	0.8535	0.4103	-0.4228	-0.2144	0.299	-0.6504	0.3129
	p	-	0.000	0.020	0.000	0.000	0.005	0.004	0.157	0.046	0.000	0.036
**FRAP**	*r*	0.9013	1.000	0.2753	0.8467	0.9177	0.4115	-0.4046	-0.3462	0.1464	-0.6159	0.2768
	p	0.000	-	0.067	0.000	0.000	0.005	0.006	0.020	0.337	0.000	0.066
**Tyrosol**	*r*	0.3449	0.2753	1.000	0.2905	0.0227	0.3517	0.2539	0.0364	0.5843	-0.0649	-0.3341
	p	0.020	0.067	-	0.053	0.882	0.018	0.092	0.812	0.000	0.672	0.025
**Verbascoside**	*r*	0.7718	0.8467	0.2905	1.000	0.7256	0.3009	-0.0832	-0.5629	0.1582	-0.4605	0.0870
	p	0.000	0.000	0.053	-	0.000	0.045	0.587	0.000	0.299	0.001	0.570
**Oleuropein**	*r*	0.8535	0.9177	0.0227	0.7256	1.000	0.2898	-0.6292	-0.3052	-0.004	-0.6693	0.4421
	p	0.000	0.000	0.882	0.000	-	0.054	0.000	0.041	0.979	0.000	0.002
**Apigenin-7-O-glucoside**	*r*	0.4103	0.4115	0.3517	0.3009	0.2898	1.000	-0.1626	0.1527	0.6162	-0.3841	0.1229
	p	0.005	0.005	0.018	0.045	0.054	-	0.286	0.317	0.000	0.009	0.421
**Luteolin**	*r*	-0.4228	-0.4046	0.2539	-0.0832	-0.6292	-0.1626	1.000	-0.1575	0.1195	0.4357	-0.6274
	p	0.004	0.006	0.092	0.587	0.000	0.286	-	0.301	0.434	0.003	0.000
**Fe**	*r*	-0.2144	-0.3462	0.0364	-0.5629	-0.3052	0.1527	-0.1575	1.000	0.3661	0.2255	-0.1833
	p	0.157	0.020	0.812	0.000	0.041	0.317	0.301	-	0.013	0.136	0.228
**Mn**	*r*	0.2990	0.1464	0.5843	0.1582	-0.004	0.6162	0.1195	0.3661	1.000	-0.0453	-0.1901
	p	0.046	0.337	0.000	0.299	0.979	0.000	0.434	0.013	-	0.768	0.211
**K**	*r*	-0.6504	-0.6159	-0.0649	-0.4605	-0.6693	-0.3841	0.4357	0.2255	-0.0453	1.000	-0.5463
	p	0.000	0.000	0.672	0.001	0.000	0.009	0.003	0.136	0.768	-	0.000
**Mg**	*r*	0.3129	0.2768	-0.3341	0.087	0.4421	0.1229	-0.6274	-0.1833	-0.1901	-0.5463	1.000
	p	0.036	0.066	0.025	0.570	0.002	0.421	0.000	0.228	0.211	0.000	-

*Relationships among the observed variables are expressed as correlation coefficients (*r*) and significance (*p*). Abbreviations: DPPH – 2,2-diphenyl-1-picrylhydrazyl, FRAP – ferric reducing ability of the plasma, Verb – Verbascoside, Ole – oleuropein, Api-7-O – apigenin-7-O-glucoside, Fe – iron, Mn – manganese, K – potassium, Mg – magnesium. (n=45)

Table S2. Correlation* between the concentrations of verbascoside, oleuropein, and potassium in leaves of Drobnica, Istarska bjelica, Leccino, Levantinka, and Oblica olive cultivars collected at different sampling times.

Source of variation		Drobnica			Istarska bjelica			Leccino			Levantinka			Oblica		
		Verb	Ole	K	Verb	Ole	K	Verb	Ole	K	Verb	Ole	K	Verb	Ole	K
**Verb**	*r*	1.000	0.9289	-0.5569	1.000	0.3162	-0.4740	1.000	0.8685	-0.613	1.000	0.9325	-0.7299	1.000	0.9798	-0.8197
	p	-	0.000	0.119	-	0.407	0.197	-	0.002	0.079	-	0.000	0.026	-	0.000	0.007
**Ole**	*r*	0.9289	1.000	-0.7641	0.3162	1.000	-0.0209	0.8685	1.000	-0.838	0.9325	1.000	-0.8457	0.9798	1.000	-0.7813
	p	0.000	-	0.017	0.4070	-	0.957	0.002	-	0.005	0.000	-	0.004	0.000	-	0.013
**K**	*r*	-0.5569	-0.7641	1.000	-0.4740	-0.0209	1.000	-0.6130	-0.8380	1.000	-0.7299	-0.8457	1.000	-0.8197	-0.7813	1.000
	p	0.119	0.017	-	0.197	0.957	-	0.079	0.005	-	0.026	0.004	-	0.007	0.013	-

*Relationships among the observed variables are expressed as correlation coefficients (*r*) and significance (*p*). Abbreviations: Verb – verbascoside, Ole – oleuropein, K – potassium. (n = 9).

Table S3. Chemical properties of the *Terra rossa* soil.

Parameter	Value
pH (H_2O)	7.89
pH (KCl)	7.08
Total N (%)	0.43
P mg $100g^{-1}$	4.98
K mg $100g^{-1}$	51.00
Organic matter (%)	7.16

Table S4. Description of olive cultivars included in the study.

	Drobnica	Istarska bjelica	Leccino	Levantinka	Oblica
1. Morphology					
1.1. Tree					
Vigor	medium[48]	strong[23]	strong[23]	strong[23]	medium[23]
Growth habit	erect[21, 48]	erect[23]	drooping[23]	spreading[23]	spreading[23]
Canopy density	medium[48]	dense[23]	dense[23]	dense[23]	sparse[23]
1.2. Leaf					
Shape	elliptic-lanceolate[48]	elliptic-lanceolate[23]	elliptic-lanceolate[23]	elliptic[23]	lanceolate[23]
Length	medium[48]	long[23]	medium[23]	long[23]	medium[23]
Width	medium[48]	medium[23]	medium[23]	broad[23]	medium[23]
1.3. Inflorescence					
Length	medium[48]	medium[23]	short[23]	medium[23]	medium[23]
Number of flowers	few[48]	medium[23]	medium[23]	medium[23]	medium[23]
1.4. Fruit					
Weight	medium[48]	medium[23]	medium[23]	medium[23]	high[23]
Shape	ovoid[48]	ovoid[23]	ovoid[23]	ovio-elongated[23]	spherical[23]
2. Agronomic characteristics					
Fertility	partially self-incompatible[14]	partially self-compatible[23]	partially self-incompatible[14]	partially self-incompatible[14]	partially self-incompatible[14]
Productivity	high, constant[24]	high, constant[23]	high, constant[23]	high, constant[23]	medium, alternate[23]
Cold tolerance	sensitive[25],very tolerant[24]	good tolerance[23]	good tolerance[23]	very sensitive[23]	good tolerance[21]

Different superscript numbers indicate cited references: [14]Vuletin Selak, G.; Perica, S.; Goreta Ban, S.; Radunić, M.; Poljak, M. Reproductive Success after Self-pollination and Cross-pollination of Olive Cultivars in Croatia. HortScience, 2011, 46, 186-191; [21]Strikić, F.; Klepo, T.; Rošin, J.; Radunić, M. *Udomaćene sorte maslina u Republici Hrvatskoj*. Institut za jadranske kulture i melioraciju krša: Split, Croatia, 2010; p. 82.; [23] Barranco, D.; Cimato, A.; Fiorino, P.; Rallo, L.; Touzani, A.; Castañeda, C.; Serafin, F.; Trujillo, I. *World catalogue of olive varieties*. International Olive Oil Council: Madrid, Spain, 2000; pp. 51-216; [24]Škarica, B.; Žužić, I.; Bonifačić, M. *Maslina i maslinovo ulje visoke kakvoće u Hrvatskoj*. Tipograf d.d.: Rijeka, Croatia, 1996; p. 315.; [25] Tomić Maksan, M.; Brečić, R. Organic olive oil in Croatia. In Sustainability of European Food Quality Schemes: Multi-Performance, Structure, and Governance of PDO, PGI, and Organic Agri-Food Systems; Arfini, F., Bellassen, V., Eds.; Springer International: Cham, Switzerland, 2019; p. 136.; [48]Brkljača, M.; Rumora, J.; Marcelić, Š.; Juranov, A. Morphological and pomological characterization of two *Olea europaea* cultivars,'Karbuncela' and 'Drobnica', grown on the island of Dugi otok, Croatia. In *VIII International Olive Symposium*, Split, Croatia, 10-16 October 2016; Perica S., Vuletin Selak G., Klepo T., Ferguson L., Sebastiani L., Eds; ISHS Acta Horticulturae: 30 April 2018; pp. 41-46;.
